# The ELF3-TRIM22-MAVS signaling axis regulates type I interferon and antiviral responses

**DOI:** 10.1128/jvi.00004-25

**Published:** 2025-03-31

**Authors:** Qiaozhi Zhao, Pan Pan, Lirong Mo, Jiangtao Wu, Shengjie Liao, Hua Lu, Qiwei Zhang, Xiaoshen Zhang

**Affiliations:** 1 The First Affiliated Hospital of Jinan University 162698, Guangzhou, Guangdong, China; 2 School of Basic Medical Science, State Key Laboratory of Respiratory Disease, Guangzhou Medical University 655509, Guangzhou, Guangdong, China; 3 Department of Basic Medicine and Public Health, Jinan University 47885, Guangzhou, Guangdong, China; 4 Department of Immunology and Microbiology, Institute of Medical Microbiology, College of Life Science and Technology, Jinan University 506476 , Guangzhou, Guangdong, China; 5 Ministry of Education, Key Laboratory of Viral Pathogenesis & Infection Prevention and Control (Jinan University) 47885, Guangzhou, Guangdong, China; University Medical Center Freiburg, Freiburg, Germany

**Keywords:** TRIM22, MAVS, NLRX1, innate immunity, IFN-β, antiviral response

## Abstract

Activation of the innate immune response is essential for host cells to restrict the dissemination of invading viruses and other pathogens. Proteins belonging to the tripartite motif (TRIM) family are key effectors in antiviral innate immunity. Among these, TRIM22, a RING-type E3 ubiquitin ligase, has been recognized as a significant regulator in the pathogenesis of various diseases. In the present study, we identified TRIM22 as a critical modulator of mitochondrial antiviral signaling protein (MAVS) activation. Loss of TRIM22 function led to reduced production of type I interferons (IFNs) in response to viral infection such as influenza A virus (IAV) or vesicular stomatitis virus (VSV), thereby facilitating viral replication. Mechanistically, TRIM22 was found to enhance retinoic acid-inducible gene I (RIG-I)-mediated signaling through the catalysis of Lys63-linked polyubiquitination of MAVS, which, in turn, activated the TANK-binding kinase 1 (TBK1)/interferon regulatory factor 3 (IRF3) pathway, driving IFN-β production. Additionally, TRIM22 was shown to inhibit the assembly of the MAVS-NLRX1 inhibitory complex, further amplifying innate immune responses. Our findings also demonstrated that RNA virus infection upregulated TRIM22 expression via the nuclear translocation of ELF3, a transcription factor that activates TRIM22 gene expression. This regulatory loop underscores the role of TRIM22 in modulating the type I IFN pathway, providing critical insights into the host’s antiviral defense mechanisms. Our research highlights the potential of targeting the ELF3-TRIM22-MAVS axis as a therapeutic strategy for enhancing antiviral immunity and preventing RNA virus infections.

**IMPORTANCE:**

Interferon (IFN)-mediated antiviral responses are crucial for the host’s defense against foreign pathogens and are regulated by various signaling pathways. The tripartite motif (TRIM) family, recognized for its multifaceted roles in immune regulation and antiviral defense, plays a significant part in this process. In our study, we explored the important role of TRIM22, a protein that helped regulate the host’s immune response to viral infections. We found that TRIM22 enhances the Lys63-linked polyubiquitination of mitochondrial antiviral signaling protein (MAVS), which was essential for producing type I interferons. Interestingly, we discovered that the expression of TRIM22 increases after an RNA virus infection, due to a transcription factor ELF3, which moved into the nucleus of cells to activate TRIM22 transcription. This created a feedback loop that strengthens the role of TRIM22 in modulating the type I IFN pathway. By uncovering these mechanisms, we aimed to enhance our understanding of how the immune system works and provide insights that could lead to innovative antiviral therapies.

## INTRODUCTION

Innate immunity represents the host’s first line of defense against invading viruses and other pathogens. During viral infection, host cells deploy pattern recognition receptors (PRRs) to detect viruses as foreign invaders by recognizing pathogen-associated molecular patterns (PAMPs), thereby initiating the innate immune response (1, 2). Among the major PRRs, cytosolic retinoic acid-inducible gene I (RIG-I)-like receptors (RLRs) play a pivotal role (3). While RIG-I detects a variety of viruses, these receptors share a common adaptor protein, mitochondrial antiviral signaling protein (MAVS) (also known as CARDIF, IPS-1, or VISA), which is localized on the outer mitochondrial membrane and is essential for downstream signal transduction (4). Upon binding to viral RNAs, RIG-I undergoes conformational changes that activate MAVS, subsequently triggering the activation of TANK-binding kinase 1 (TBK1) and interferon regulatory factor 3 (IRF3), leading to the production of type I interferons (IFNs) and other antiviral cytokines crucial for limiting viral infection (5, 6).

In humans, the tripartite motif (TRIM) protein family comprises over 80 members, featuring RING domains that enable them to function as ubiquitin E3 ligases, mediating the ubiquitination of target proteins (7). The role of TRIM proteins in innate immune signaling pathways is largely attributed to their capacity to catalyze ubiquitination, primarily through Lys48 (K48)- or Lys63 (K63)-linked polyubiquitination (8–10). In this study, we observed a significant induction of TRIM22 (also referred to as GPSTAF50, RNF94, or STAF50) expression in response to RNA virus infection. Our experiments revealed that viral infection promoted the nuclear translocation of ELF3, a member of the ETS transcription factor family, thereby enhancing TRIM22 expression and contributing to its antiviral effects (11). ELF3 (also known as ESE-1, ESX, EPR-1, and ERT) is a transcription factor that plays a critical regulatory role by binding to specific DNA sequences to activate or repress gene expression (12). We identified TRIM22 as a positive regulator of the RIG-I-mediated innate immune pathway during RNA virus infection, acting through its interaction with MAVS to promote K63-linked ubiquitination.

Nucleotide-binding oligomerization domain-like receptor X1 (NLRX1), another mitochondrial outer membrane protein, is known as a negative regulator of the innate immune response to viral infection (13, 14). Our study further demonstrated that TRIM22 disrupts the formation of the NLRX1-MAVS complex, thereby relieving the negative regulation of the RIG-I-MAVS signaling pathway. These findings elucidate a critical mechanism by which TRIM22 modulates innate immunity and exerts antiviral activity. Our research provides compelling evidence for the central role of the TRIM22-driven MAVS signaling pathway in the pathogenesis of RNA virus infection and suggests that the TRIM22-MAVS axis could serve as a promising therapeutic target for the treatment of RNA virus infections.

## RESULTS

### TRIM22 is induced by RNA virus infection and exerts antiviral effects

**Fig 1:**
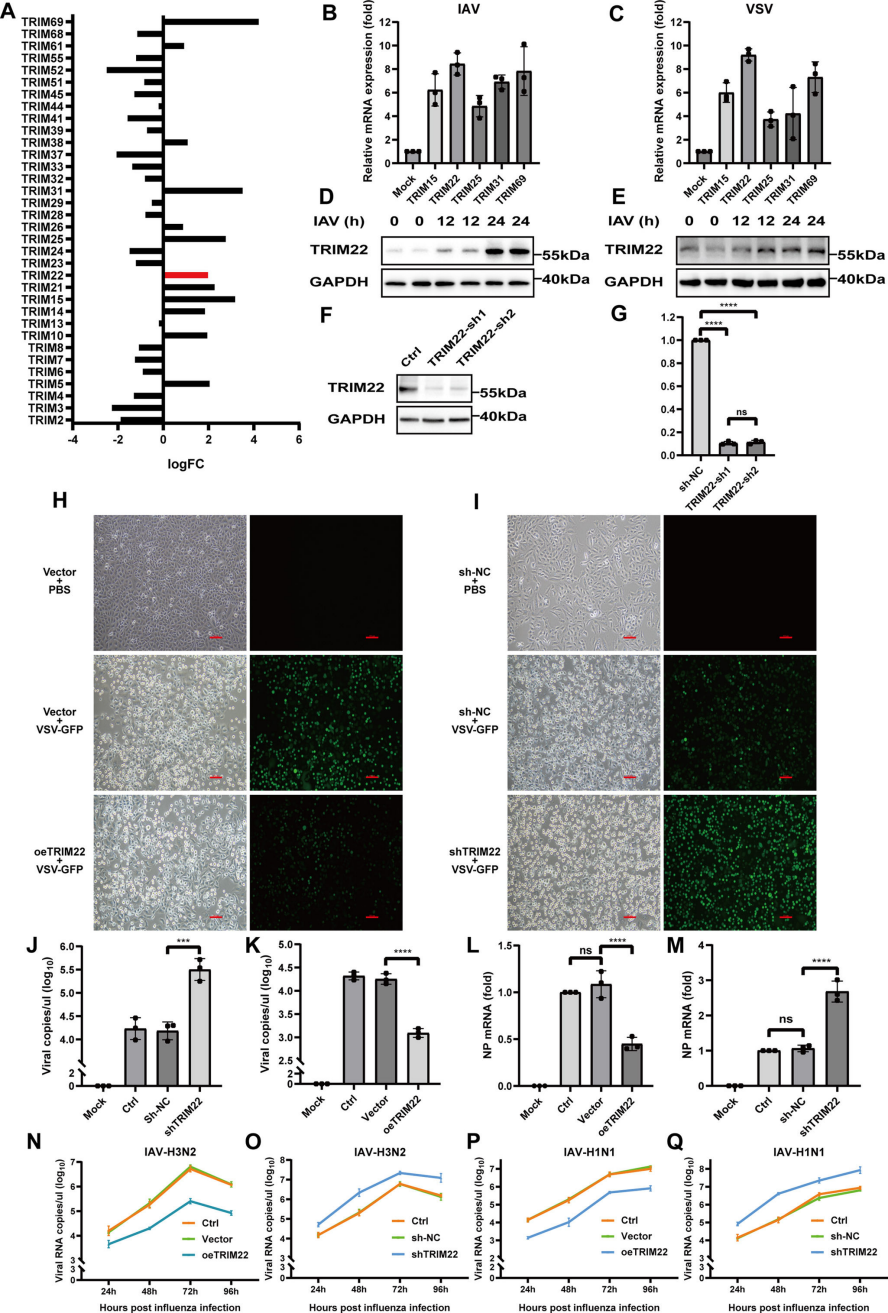
TRIM22 is induced by RNA virus infection and exerts antiviral effects. (A) TRIM family mRNA expression in human airway epithelial cells in response to influenza from the GEO database. (B and C) Real-time quantitative reverse transcription-PCR (RT-qPCR) measuring the expression levels of TRIM15, TRIM22, TRIM25, TRIM31, and TRIM69 in cells infected with IAV (H1N1) (MOI = 0.1) (B) and VSV (MOI = 0.1) (C) for 24 h. (D and E) Western blot analysis of TRIM22 expression in A549 (D) and BEAS-2B (E) cells infected with IAV (H1N1) (MOI = 0.1) at each time point. (F and G) Western blot (F) and RT-qPCR (G) measuring TRIM22 gene expression in A549 clones silenced for TRIM22, TRIM22-sh1, and TRIM22-sh2 compared to control cells (sh-NC). (H and I) Fluorescence microscopy of VSV-GFP (MOI = 0.1) replication in A549 cells transfected with overexpressed TRIM22 (H) or in TRIM22 knockdown A549 cells (I) infected for 24 h (bright-field, left panel; fluorescence, right panel). Scale bars, 100 µm. (J and K) Overexpression of TRIM22 (K) or TRIM22 knockdown (J) A549 cells were infected with IAV (PR8) (MOI = 0.1) for 24 h. The IAV genome was extracted from the cell culture supernatant, and the viral copy number was measured by qPCR using a standard curve with the IAV NP gene plasmid. (L and M) Overexpression of TRIM22 (M) or shTRIM22 (L) A549 cells were infected with IAV (H1N1) (MOI = 0.1) for 24 h. RNA was extracted, and IAV NP mRNA was measured by RT-qPCR. (N–Q) Kinetics of viral replication of IAV-H3N2 (MOI = 0.05) (N and O) or IAV-H1N1 (MOI = 0.05) (P and Q) isolates up to 96 h post-infection in overexpression of TRIM22 or TRIM22 knockdown A549 cells. A one-way analysis of variance (ANOVA) test was used for panels B, C, G, and J–M. All data are representative of three independent experiments with three biological replicates and are presented as mean ± S.D. (ns, nonsignificant; *P < 0.05; ***P < 0.001; ****P < 0.0001).

We initially analyzed public data sets from the Gene Expression Omnibus (GEO) and observed that the expression of TRIM family proteins was upregulated in response to RNA viruses, as indicated in data set GSE32139 (15, 16) (Fig. 1A). During our screening of TRIM family proteins in A549 cells following infection with influenza A virus (IAV) or vesicular stomatitis virus (VSV), we observed that TRIM22 was the most prominently upregulated (Fig. 1B and C). Further investigation revealed that IAV infection led to a marked increase in TRIM22 expression at 24 h post-infection in both A549 and BEAS-2B cells (Fig. 1D and E). To further confirm the antiviral capability of TRIM22, we investigated its role in innate immunity by knocking down TRIM22 using short hairpin RNA (shRNA) in human A549 cells. This knockdown effectively reduced TRIM22 levels at both the protein and mRNA levels (Fig. 1F and G). We then chose the TRIM22-sh1 for the subsequent experiments. A549 cells were transfected with exogenous TRIM22 and then infected with green fluorescent protein (GFP)-expressing vesicular stomatitis virus (VSV-GFP). Fluorescence microscopy revealed that TRIM22 overexpression inhibited viral replication compared to cells transfected with a control vector (Fig. 1H). In contrast, knockdown of TRIM22 resulted in increased viral replication (Fig. 1I). Furthermore, we quantified the IAV viral copy numbers in the cell supernatant. We found that the viral load was significantly lower in the culture medium of cells overexpressing TRIM22 compared to the control group (Fig. 1K). Conversely, knockdown of TRIM22 led to a marked increase in viral copy numbers (Fig. 1J). Overexpression of TRIM22 also reduced the amount of NP protein in cells following IAV infection, whereas knockdown of TRIM22 increased IAV replication (Fig. 1L and M). To observe the *in vitro* replication kinetics of the IAV-H1N1 (Fig. 1N and O) and IAV-H3N2 (Fig. 1P and Q), individual viruses were infected at MOI of 0.05 in overexpression of TRIM22 or TRIM22 knockdown A549 cells, and viral genome copies were quantified at various time points during the infection using quantitative PCR (qPCR). Comparisons of the resulting IAV genome copies provide strong evidence that TRIM22 can inhibit viral replication.

### TRIM22 regulates TBK1/IRF3-dependent IFN-β expression

**Fig 2:**
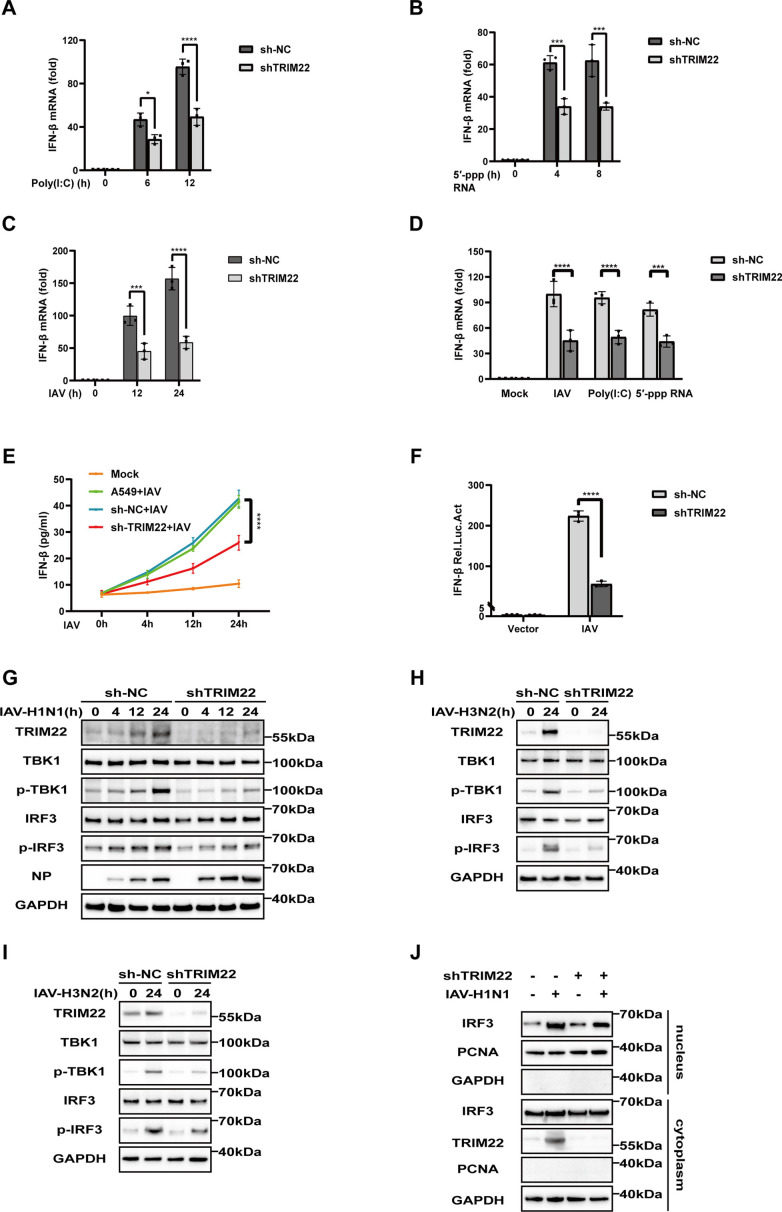
TRIM22 regulates TBK1/IRF3-dependent IFN-β expression. (A–C) RT-qPCR analysis of IFN-β mRNA in TRIM22 knockdown or sh-NC A549 cells treated with poly(I:C) (A), 5′-pppRNA (B), or infected with IAV-H1N1 (MOI = 0.1) (C) at each time point. (D) RT-qPCR analysis of IFN-β mRNA in TRIM22 knockdown A549 cells untreated or infected with IAV-H1N1 (MOI = 0.1) or treated with poly(I:C), 5′-pppRNA for 12 h. (E) ELISA analysis of IFN-β in the cell culture supernatant of wild type, TRIM22 knockdown, or sh-NC A549 cells infected with IAV (MOI = 0.1) at each time point. (F) IFN-β promoter luciferase reporter assay of HEK293T-shTRIM22 or shNC cells stimulated with IAV (MOI = 0.1) for 24 h. Results are expressed relative to Renilla luciferase activity, with relative luciferase activity determined by calculating the ratio of firefly luciferase to Renilla luciferase. (G) Western blot analysis of phosphorylated and total IRF3, TBK1, and IAV NP protein in lysates of TRIM22 knockdown or sh-NC A549 cells infected with IAV-H1N1 (MOI = 0.1) at each time point. (H) Western blot analysis of phosphorylated and total IRF3 and TBK1 protein in lysates of TRIM22 knockdown or sh-NC A549 cells infected with IAV-H3N2 (MOI = 0.1) at each time point. (I) Western blot analysis of phosphorylated and total IRF3 and TBK1 protein in lysates of TRIM22 knockdown or sh-NC BESA-2B cells infected with IAV-H3N2 (MOI = 0.1) at each time point. (J) Western blot analysis of IRF3 in the cytoplasmic and nuclear fractions of TRIM22 knockdown or sh-NC A549 cells infected with IAV-H1N1 (MOI = 0.1) for 24 h. A two-way ANOVA test was used for panels A–F. All data are representative of three independent experiments with three biological replicates and are presented as mean ± S.D. (ns, nonsignificant; *P < 0.05; ***P < 0.001; ****P < 0.0001).

To investigate the mechanism by which TRIM22 exerts its antiviral effects, we focused on the role of type I IFN signaling in antiviral defense (17, 18). We stimulated A549 cells with IAV-H1N1, 5′-triphosphorylated RNA (5′pppRNA, the ligand of RLRs), or dsRNA poly(I:C) (high molecular weight poly I:C, the ligand of RLRs), and subsequently measured IFN-β mRNA levels. Our results indicated that IFN-β expression following stimulation with poly(I:C), 5′pppRNA, or IAV-H1N1 infection was significantly reduced in TRIM22 knockdown cells compared to wild-type (WT) cells (Fig. 2A through D). We further analyzed the levels of IFN-β protein in the supernatant of IAV-H1N1-infected cells at various time points using an enzyme-linked immunosorbent assay (ELISA). The results showed a marked decrease in IFN-β secretion in TRIM22 knockdown cells compared to controls (Fig. 2E), indicating that knockdown of TRIM22 significantly impairs both the mRNA and protein expression of IFN-β following IAV-H1N1 infection. Given the crucial role of TRIM proteins in mediating antiviral innate immunity, we further explored the role of TRIM22 in innate immune signaling. The luciferase assay revealed that knockdown of TRIM22 significantly reduced IAV-H1N1-induced activation of the IFN-β promoter (Fig. 2F). Since IFN-β production depends on the activation of the MAVS signaling pathway, we assessed the impact of TRIM22 on key signaling molecules, including TBK1 and IRF3, in A549 cells treated with IAV-H1N1 at various time points. Consistent with expectations, IAV-H1N1 infection induced the phosphorylation of IRF3 and TBK1 in A549 cells. However, in TRIM22 knockdown cells, the phosphorylation of TBK1 and IRF3 was significantly inhibited, and IAV NP protein levels increased in response to IAV-H1N1 infection (Fig. 2G). A similar effect was observed in TRIM22 knockdown A549 (Fig. 2H) and BESA-2B (Fig. 2I) cell lines following IAV-H3N2 infection. Moreover, TRIM22 knockdown cells reduced translocation of IRF3 to the nucleus compared to WT cells following IAV-H1N1 infection (Fig. 2J). Collectively, these data demonstrate that TRIM22 positively regulates type I interferon signaling in antiviral innate immunity.

### The PRY/SPRY domain of TRIM22 interacts with the proline-rich domain of MAVS

**Fig 3:**
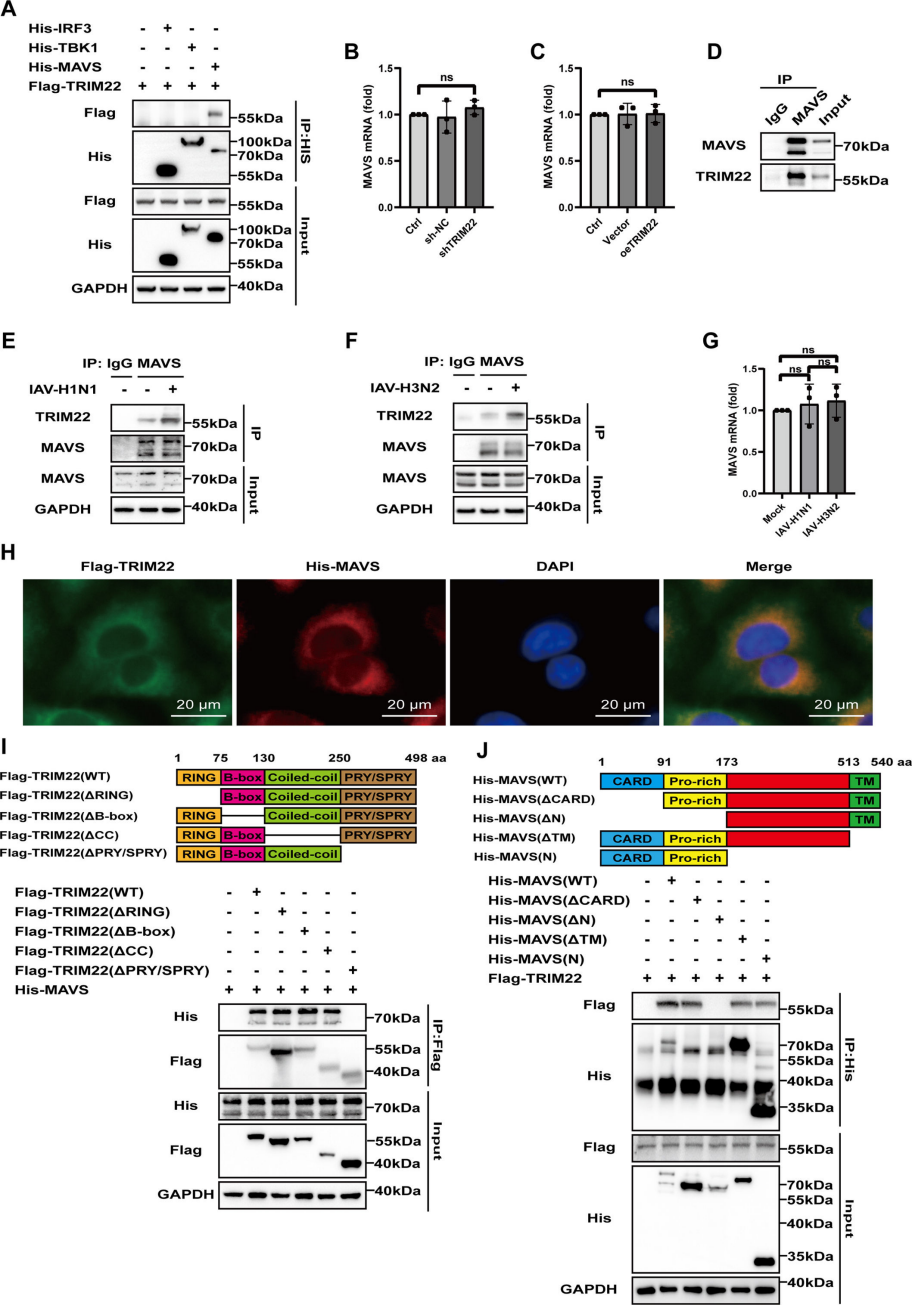
The PRY/SPRY domain of TRIM22 interacts with the proline-rich domain of MAVS. (A) Western blot analysis using anti-Flag or anti-His antibodies for proteins that co-immunoprecipitated with Flag-tagged TRIM22 from lysates of HEK293T cells that were also co-transfected with plasmids encoding His-tagged IRF3, TBK1, or MAVS. Input represents western blot analysis of whole-cell lysates without IP. (B) RT-qPCR analysis of MAVS mRNA in TRIM22 knockdown or sh-NC A549 cells. (C) RT-qPCR analysis of MAVS mRNA in TRIM22 overexpression or vector A549 cells. (D) Co-immunoprecipitation analysis to assess the interaction of endogenous TRIM22 with MAVS in A549 cells. (E and F) Co-immunoprecipitation analysis to assess the interaction of endogenous TRIM22 with MAVS in A549 cells infected with IAV-H1N1 or IAV-H3N2 (MOI = 0.1) for 24 h. Input represents western blot analysis of whole-cell lysates without IP. (G) RT-qPCR analysis of MAVS mRNA in A549 cells infected with IAV (MOI = 0.1) for 24 h. (H) Fluorescence microscopy of A549 cells transfected for 48 h with plasmids expressing Flag-TRIM22 and His-MAVS, followed by labeling of TRIM22 and MAVS with a Flag- or His-specific primary antibody and an Alexa Fluor 568-conjugated goat anti-mouse IgG secondary antibody (red) and an Alexa Fluor 488-conjugated goat anti-rabbit IgG secondary antibody (green). Scale bars: 20 µm. (I) Binding of Flag-TRIM22 or its truncation mutants with His-MAVS in co-transfected HEK293T cells, as determined by co-immunoprecipitation and western blot analysis. Flag-TRIM22 domains and truncation mutants are shown on top. (J) Binding of His-MAVS or its truncation mutants with Flag-TRIM22 in co-transfected HEK293T cells, as determined by co-immunoprecipitation and western blot analysis. His-MAVS domains and truncation mutants are shown on top. A one-way ANOVA test was used for panels B, C, and F. All data are representative of three independent experiments with three biological replicates and are presented as mean ± S.D. (ns, nonsignificant; *P < 0.05; ***P < 0.001; ****P < 0.0001).

The production of IFN-β is dependent on the activation of the MAVS signaling pathway (19, 20). To elucidate the specific pathway through which TRIM22 regulates IFN-β signaling, a co-immunoprecipitation (co-IP) assay was designed to assess the interaction between Flag-tagged TRIM22 and key adaptor proteins, including His-tagged MAVS, His-tagged TBK1, and His-tagged IRF3. The results demonstrated that MAVS could interact with TRIM22, whereas no interaction was observed between TBK1 or IRF3 and TRIM22 (Fig. 3A). Importantly, neither the deficiency (Fig. 3B) nor the overexpression (Fig. 3C) of TRIM22 affected the mRNA level of MAVS. Further co-IP assays confirmed the endogenous interaction between MAVS and TRIM22 (Fig. 3D). Notably, this interaction was significantly enhanced after IAV-H1N1 (Fig. 3E) or IAV-H3N2 (Fig. 3F) infection, without altering the mRNA level of MAVS (Fig. 3G), suggesting that TRIM22 promoted the antiviral response through interaction with MAVS. Immunofluorescence experiments proved that TRIM22 and MAVS could co-localize in the cytoplasm (Fig. 3H). TRIM22 consists of a RING finger domain, a B-box domain, a coiled-coil region, and a PRY/SPRY domain (11). To identify which domain of TRIM22 is crucial for its interaction with MAVS, we generated several TRIM22 deletion mutants and tested them using co-IP assays. The results showed that deletion of the C-terminal PRY/SPRY domain abolished the interaction with MAVS, while the PRY/SPRY domain alone was sufficient to mediate this interaction (Fig. 3I). MAVS is composed of a caspase activation and recruitment domain, a proline-rich domain, and a mitochondrial transmembrane domain. To determine which MAVS domains are involved in binding TRIM22, we generated four MAVS deletion mutants, each lacking one specific domain (21). Co-IP assays in HEK293T cells revealed that deletion of the MAVS proline-rich domain disrupted its interaction with TRIM22, demonstrating that the proline-rich domain alone was sufficient to facilitate the association with TRIM22 (Fig. 3J). In summary, these findings demonstrate that TRIM22 interacts with MAVS, and intracellular RNA virus infection promotes this interaction.

### TRIM22 promotes the K63-linked polyubiquitination of MAVS

**Fig 4:**
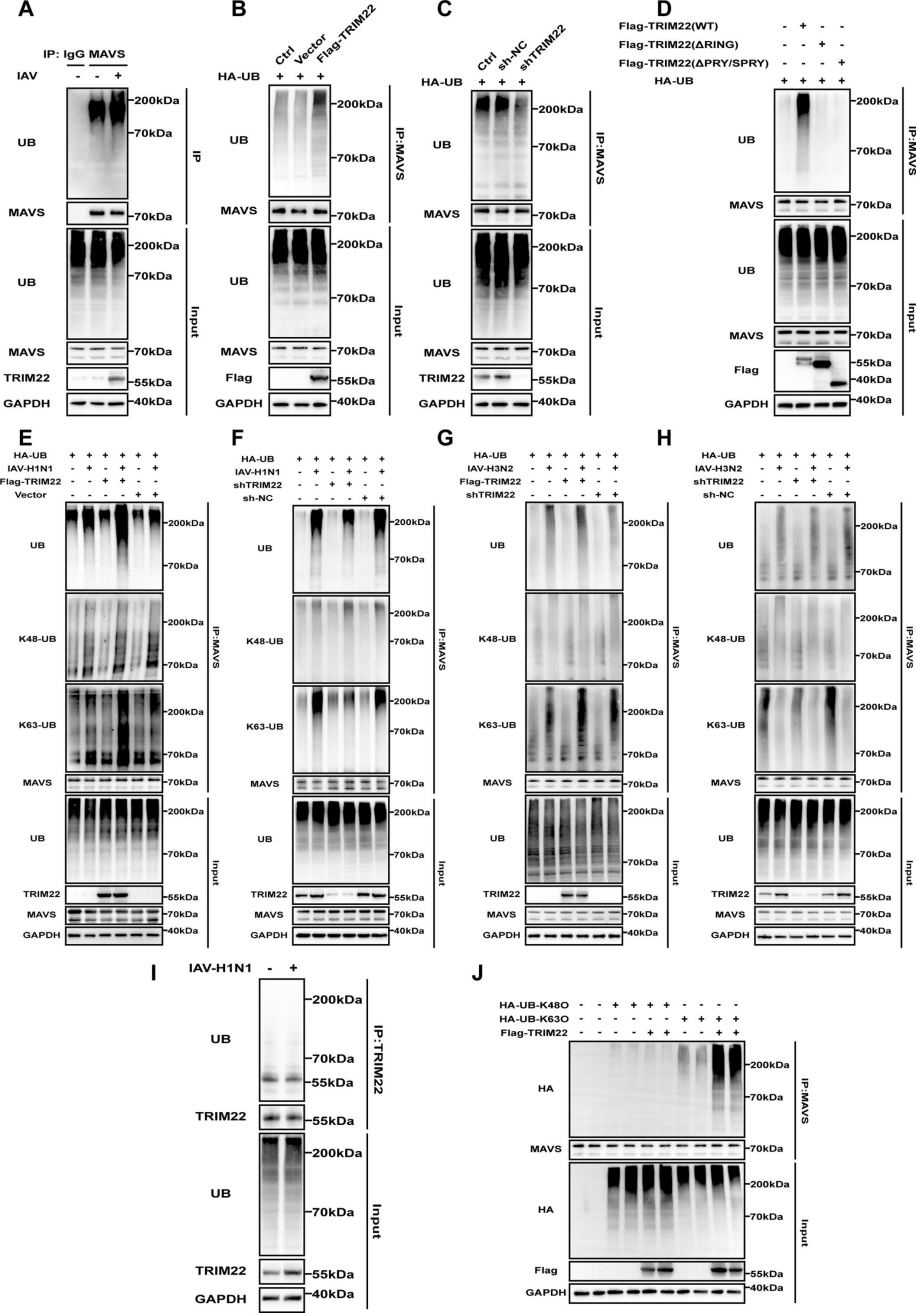
TRIM22 promotes the K63-linked polyubiquitination of MAVS. (A) Co-immunoprecipitation analysis to assess endogenous MAVS ubiquitination in A549 cells infected with IAV (MOI = 0.1) for 24 h. (B) Co-immunoprecipitation analysis to assess endogenous MAVS ubiquitination in overexpression TRIM22 or vector HEK293T cells transfected with HA-ubiquitin. (C) Co-immunoprecipitation analysis to assess endogenous MAVS ubiquitination in HEK293T-shTRIM22 or sh-NC cells expressing HA-ubiquitin. (D) Flag-TRIM22 or its truncation mutants and HA-ubiquitin were co-transfected in HEK293T cells, as determined by co-immunoprecipitation and Western blot analysis to assess endogenous MAVS ubiquitination. (E) Co-immunoprecipitation analysis to assess endogenous MAVS ubiquitination in overexpression TRIM22 or vector HEK293T cells infected with IAV-H1N1 (MOI = 0.1) for 24 h expressing HA-ubiquitin. (F) Co-immunoprecipitation analysis to assess endogenous MAVS ubiquitination in HEK293T-shTRIM22 or sh-NC cells infected with IAV-H1N1 (MOI = 0.1) for 24 h expressing HA-ubiquitin. (G) Co-immunoprecipitation analysis to assess endogenous MAVS ubiquitination in overexpression TRIM22 or vector HEK293T cells infected with IAV-H3N2 (MOI = 0.1) for 24 h expressing HA-ubiquitin. (H) Co-immunoprecipitation analysis to assess endogenous MAVS ubiquitination in HEK293T-shTRIM22 or sh-NC cells infected with IAV-H3N2 (MOI = 0.1) for 24 h expressing HA-ubiquitin. (I) Co-immunoprecipitation analysis to assess endogenous TRIM22 ubiquitination in A549 cells infected with IAV-H1N1 (MOI = 0.1) for 24 h. (J) Co-immunoprecipitation analysis to assess endogenous MAVS ubiquitination in HEK293T cells co-transfected with Flag-TRIM22, with or without HA-ubiquitin (K63O) or HA-ubiquitin (K48O). All data are representative of three independent experiments with three biological replicates.

K63-linked ubiquitination of MAVS is essential for the induction of type I interferon during the innate immune response (21, 22). Our results demonstrated that infection with IAV-H1N1 led to an increase in the ubiquitination of MAVS along with promoted expression of TRIM22 (Fig. 4A). TRIM22, a RING-type E3 ubiquitin ligase with known ubiquitination activity, was shown by our results to increase the ubiquitination of endogenous MAVS in HEK293T cells upon overexpression (Fig. 4B). We further verified that the knockdown of TRIM22 reduced the ubiquitination of endogenous MAVS (Fig. 4C). Additionally, the RING and PRY/SPRY domains were sufficient to abolish the E3 ligase activity of TRIM22 in catalyzing the polyubiquitination of MAVS in HEK293T cells co-transfected with Flag-TRIM22 or its Del-RING and Del-PRY/SPRY mutants (Fig. 4D). To explore whether TRIM22 modulates MAVS ubiquitination under viral infection conditions, we employed IAV-infected cells with either overexpression or knockdown of TRIM22. As shown, TRIM22 enhanced the ubiquitination of MAVS following IAV-H1N1 or H3N2 infection (Fig. 4E and G), whereas TRIM22 knockdown reduced the ubiquitination levels of MAVS (Fig. 4F and H), underscoring the critical role of TRIM22 in viral infection. Furthermore, we observed no difference in the autoubiquitination levels of TRIM22 with or without IAV-H1N1 infection (Fig. 4I). To ascertain whether the TRIM22-mediated polyubiquitination of MAVS involved K48- or K63-linked ubiquitin chains, we assessed MAVS ubiquitination using K48- and K63-linkage-specific polyubiquitin antibodies. The results revealed that in TRIM22-overexpressing cells, endogenous MAVS was prominently ubiquitinated with K63-linked ubiquitin chains compared to control cells, with no detectable change in K48-linked ubiquitination (Fig. 4J). This indicates that TRIM22 selectively promotes K63-linked, but not K48-linked, polyubiquitination of MAVS. To further validate the involvement of K63-linked ubiquitination of MAVS by TRIM22, we co-transfected HEK293T cells with MAVS-Flag and either a K63-only or K48-only ubiquitin mutant. Co-immunoprecipitation analyses demonstrated that TRIM22 enhanced MAVS ubiquitination in the presence of the K63-only ubiquitin mutant, but not with the K48-only ubiquitin mutant (Fig. 4E). These findings conclusively demonstrated that TRIM22 catalyzes the K63-linked polyubiquitination of MAVS. The TRIM22-mediated K63-linked polyubiquitination of MAVS likely plays a pivotal role in modulating MAVS interactions within innate immune signaling pathways, particularly in the activation of IFN-β.

### TRIM22 promotes the dissociation of MAVS from NLRX1

**Fig 5:**
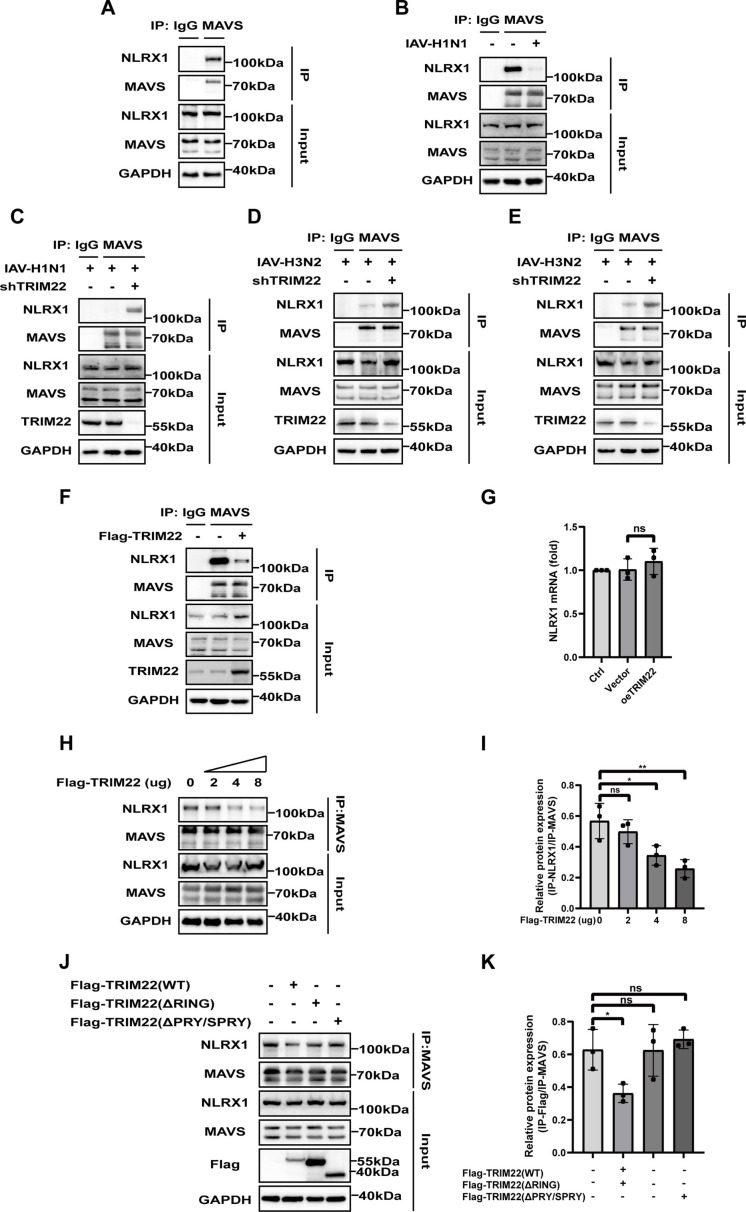
TRIM22 promotes the dissociation of MAVS from NLRX1. (A) Co-immunoprecipitation analysis to assess the interaction of endogenous NLRX1 with MAVS in A549 cells. (B) Co-immunoprecipitation analysis to assess the interaction of endogenous NLRX1 with MAVS in A549 cells infected with IAV-H1N1 (MOI = 0.1) for 24 h. (C) Co-immunoprecipitation analysis to assess the interaction of endogenous NLRX1 with MAVS in TRIM22 knockdown or sh-NC A549 cells infected with IAV-H1N1 (MOI = 0.1) for 24 h. (D) Co-immunoprecipitation analysis to assess the interaction of endogenous NLRX1 with MAVS in TRIM22 knockdown or sh-NC A549 cells infected with IAV-H3N2 (MOI = 0.1) for 24 h. (E) Co-immunoprecipitation analysis to assess the interaction of endogenous NLRX1 with MAVS in TRIM22 knockdown or sh-NC BESA-2B cells infected with IAV-H3N2 (MOI = 0.1) for 24 h. (F) Co-immunoprecipitation analysis to assess the interaction of endogenous NLRX1 with MAVS in TRIM22 overexpression or vector A549 cells. (G) RT-qPCR analysis of NLRX1 mRNA in TRIM22 overexpression or vector A549 cells. (H and I) Co-immunoprecipitation (I) and grayscale (J) analysis to assess the interaction of endogenous NLRX1 with MAVS in HEK293T cells co-transfected with increasing amounts of Flag-TRIM22. (J and K) Co-immunoprecipitation (K) and grayscale (L) analysis to assess the interaction of endogenous NLRX1 with MAVS in HEK293T cells co-transfected with Flag-TRIM22 or its Del-RING and Del-PRY/SPRY mutants. A one-way ANOVA test was used for panels I, J, and K. All data are representative of three independent experiments with three biological replicates and are presented as mean ± S.D. (ns, nonsignificant; *P < 0.05; ***P < 0.001; ****P < 0.0001).

Although MAVS plays a crucial role in antiviral immunity (23), its regulation within the mitochondria remains poorly understood. Human NLRX1, a highly conserved member of the nucleotide-binding domain and leucine-rich repeat-containing family (NLR), is known to localize to the mitochondrial outer membrane and interact with MAVS (13, 24, 25) (Fig. 5A). Previous studies have demonstrated that NLRX1 attenuates MAVS-mediated signaling pathway during HCV infection (25). In the context of IAV-H1N1 infection, we observed that the binding affinity between MAVS and NLRX1 significantly decreased compared to the control group (Fig. 5B). However, the decrease in binding affinity was restored in shTRIM22 A549 or BESA-2B cells following infection with IAV-H1N1 or IAV-H3N2 (Fig. 5C through E). To further investigate the mechanism underlying TRIM22’s role in enhancing antiviral signaling, we examined the effect of NLRX1 on the endogenous interaction with MAVS. Immunoprecipitation assays revealed a strong association between endogenous NLRX1 and MAVS, but this interaction diminished upon the overexpression of TRIM22 (Fig. 5F). Notably, overexpression of TRIM22 did not affect the mRNA or protein levels of NLRX1 (Fig. 5G). Additionally, as the expression of exogenous TRIM22 increased, the binding between NLRX1 and MAVS progressively decreased (Fig. 5H and I). Furthermore, as shown in HEK293T cells transfected with Flag-TRIM22 or its Del-RING and Del-PRY/SPRY mutant, full-length TRIM22 significantly reduced the binding affinity between NLRX1 and MAVS (Fig. 5J and K). In summary, our findings demonstrate that TRIM22 enhances the expression of interferon-β by promoting the dissociation of NLRX1 from MAVS and facilitating the K63-linked ubiquitination of MAVS.

### ELF3 transcriptionally regulates TRIM22 expression, thereby promoting the secretion of IFN-β

**Fig 6:**
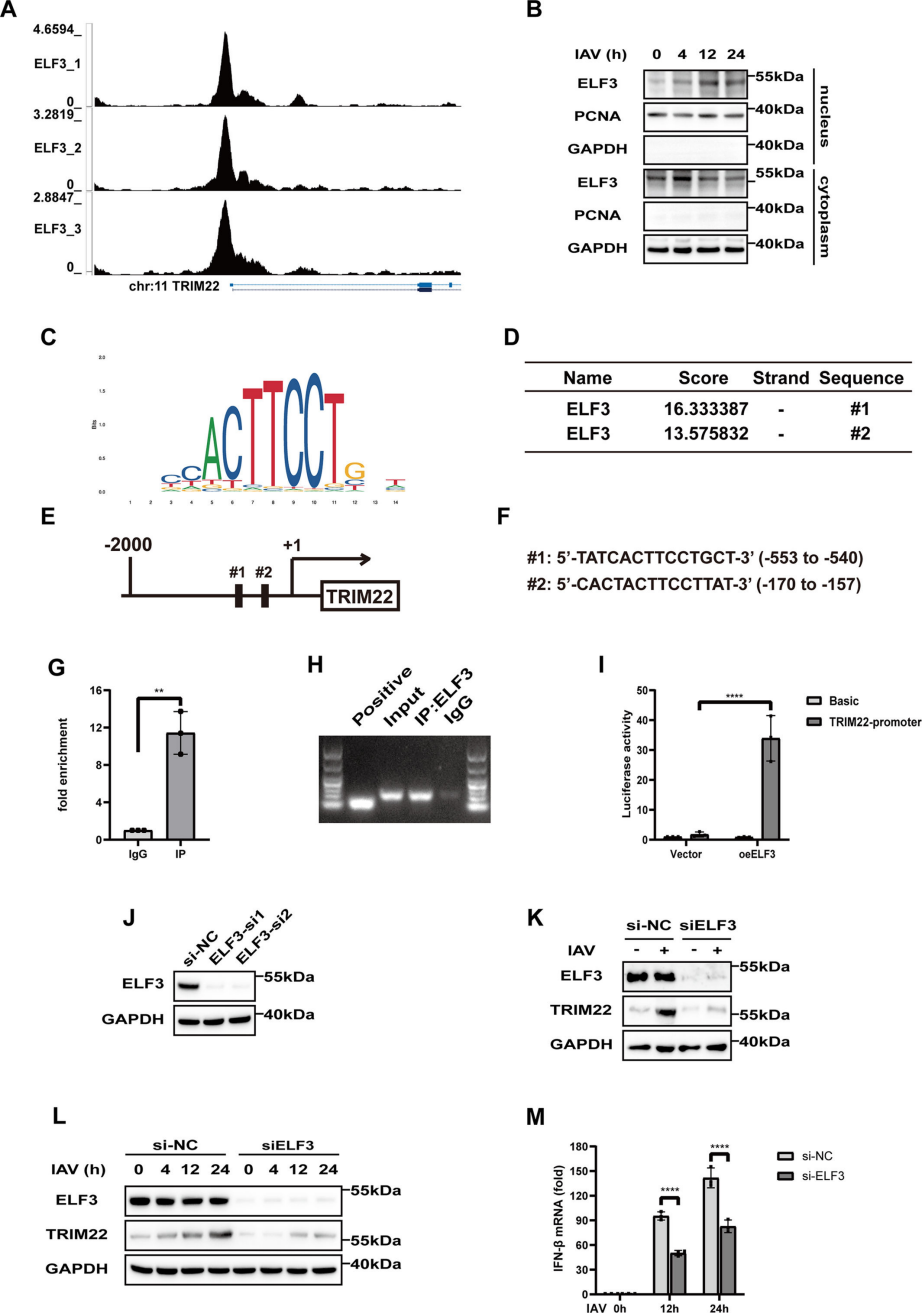
ELF3 transcriptionally regulated TRIM22 expression, thereby promoting the secretion of IFN-β. (A) ChIP-seq data from UCSC displayed coverage for ELF3 across the genome of TRIM22. (B) Western blot analysis of ELF3 in the cytoplasmic and nuclear fractions of A549 cells infected with IAV-H1N1 (MOI = 0.1) at each time point. (C) JASPAR prediction matrix for ELF3 transcription factor DNA binding sequence. (D) JASPAR-predicted binding sites of ELF3 within the TRIM22 gene regulatory region, including location, strand, and relative binding score. (E) TRIM22 transcription regulatory region and the gene. JASPAR analysis was performed from −2,000 bp to +1 bp around the transcription starting site of the TRIM22 gene. (F) Putative binding sites for the TRIM22 promoter were determined using JASPAR. (G) A ChIP assay was used to detect the TRIM22 promoter region bound by ELF3. A549 cells underwent immunoprecipitation using an anti-ELF3 antibody (IP group) or rabbit IgG (negative control group). Chromatin samples taken prior to immunoprecipitation served as the input control. RT-qPCR was then conducted to amplify and quantify the TRIM22 promoter regions in both the IP and control groups. (H) The qPCR products from (G) were further validated through gel electrophoresis. The results included marker (100–1,000 bp), lane 1: positive control (721 bp), lane 2: input control, lane 3: immunoprecipitation samples using primers targeting the TRIM22 promoter, and lane 4: rabbit IgG (negative control group). (I) Luciferase activity was detected using a dual-luciferase reporter assay to evaluate the interaction between ELF3 and the TRIM22 promoter. The pcDNA3.1-ELF3 plasmid or vector was co-transfected with firefly luciferase reporter plasmids containing the TRIM22 promoter sequence or the empty pGL3-basic vector, along with a renilla luciferase plasmid in HEK293T cells. The pGL3-Basic vector served as a negative control in each group. Relative luciferase activity was determined by calculating the ratio of firefly luciferase to renilla luciferase. (J) Western blot analysis of ELF3 in A549 cells transfected for 48 h with control siRNA (si-NC), siRNA targeting ELF3-si1, or ELF3-si2. (K) Western blot analysis of ELF3 and TRIM22 in A549 cells transfected for 48 h with si-NC and siELF3, followed by infection with IAV (MOI = 0.1) for 24 h. (L) Western blot analysis of ELF3 and TRIM22 in A549 cells transfected for 48 h with control si-NC and siELF3, followed by infection with IAV (MOI = 0.1) at different time points. (M) RT-qPCR analysis of IFN-β mRNA in A549 cells transfected for 48 h with si-NC and siELF3, followed by infection with IAV (MOI = 0.1) at each time point. A *t*-test was used for panel G. A two-way ANOVA test was used for panels I and M. All data are representative of three independent experiments with three biological replicates and are presented as mean ± S.D. (ns, nonsignificant; *P < 0.05; ***P < 0.001; ****P < 0.0001).

To explore the reasons for the upregulation of TRIM22 expression following RNA virus infection, we examined potential regulatory factors through the UCSC Genome Browser (26, 27) and identified ELF3 as a candidate transcription factor that may influence TRIM22 expression from GSM1574273 (28) (Fig. 6A). Furthermore, we observed increased translocation of ELF3 to the nucleus in A549 cells following IAV-H1N1 infection (Fig. 6B). To investigate the relationship between ELF3 and TRIM22, we predicted ELF3 binding sites on the TRIM22 gene promoter using the JASPAR database (29) (Fig. 6C through F). We then assessed whether ELF3 could indeed function as a transcription factor for TRIM22. Chromatin immunoprecipitation (ChIP) and luciferase reporter assays were conducted to determine how ELF3 binds to the TRIM22 promoter and regulates its expression. ChIP analysis using primers targeting the TRIM22 promoter (Fig. 6G) and agarose gel images using ChIP-qPCR products (Fig. 6H) revealed that ELF3 strongly binds to the TRIM22 promoter, while no binding was observed in the negative control IgG immunoprecipitation. Following confirmation that exogenous ELF3 binds to the full length of the TRIM22 promoter, we performed additional luciferase reporter assays with the TRIM22 promoter. Consistent with the ChIP analysis results, luciferase activity was significantly increased, indicating that ELF3 upregulates TRIM22 expression by binding to its promoter (Fig. 6I). To further validate the role of ELF3 in regulating TRIM22, we designed specific siRNAs targeting ELF3 and confirmed the effect of silencing ELF3 in cells; we chose ELF3-si1 for the next experiment (Fig. 6J). Notably, TRIM22 expression was suppressed in ELF3-silenced cells after IAV infection (Fig. 6K). While the expression of TRIM22 was promoted with IAV infection in a time-independent manner, silencing ELF3 resulted in the suppression of TRIM22 expression (Fig. 6L) and led to a reduction in interferon production (Fig. 6M). These findings suggest that IAV infection promotes the nuclear accumulation of ELF3, which in turn acts as a transcription factor to enhance TRIM22 expression.

## DISCUSSION

We have identified TRIM22, a member of the TRIM family, as a positive regulator of the IFN-β antiviral pathway. We demonstrated that TRIM22 targets MAVS for K63-linked polyubiquitination, thereby activating type I interferon signaling. These findings suggest a novel regulatory mechanism in the pathogenesis and prognosis of acute RNA virus infections, with potential therapeutic implications (30, 31). The innate immune system employs PRRs to recognize conserved PAMPs of invading pathogens (32, 33). Among these PRRs, RIG-I is well-characterized, recognizing cytoplasmic viral RNA and triggering innate immune signaling cascades, leading to the secretion of type I IFN (33). IFN-mediated antiviral responses are vital to the host’s defense capability against foreign pathogens (34). MAVS, a key adaptor in the RIG-I signaling pathway, is centrally located within these pathways (4, 35). Recent studies have shown that K63-linked ubiquitination of MAVS is crucial for the activation of innate immunity (21, 36), this ubiquitination recruits TBK1, leading to IRF3 activation and subsequent IFN production (37, 38). However, K48-linked ubiquitination facilitates the degradation of MAVS, thereby preventing the phosphorylation of TBK1 and IRF3, which in turn attenuates the activation of downstream antiviral signaling pathways (39).

The TRIM family, known for its diverse roles in immune regulation and antiviral defense, has been extensively studied in relation to MAVS and the RIG-I pathway. For example, TRIM25, a TRIM family member, exhibited antiviral activity (40–42), has been shown to ubiquitinate RIG-I (43), thereby promoting its activation and enhancing downstream antiviral signaling. Additionally, TRIM38 has been shown to negatively regulate RIG-I activation, thereby modulating inflammatory responses during viral infections (44). These studies highlight the multifaceted role of TRIM proteins in regulating innate immune signaling, particularly in response to RNA virus infection. Our study provides several lines of evidence that TRIM22 is a critical regulator of RIG-I-mediated antiviral immunity through the direct conjugation of K63-linked ubiquitin chains to MAVS. Consistent with this, cells lacking TRIM22 were unable to catalyze K63-linked ubiquitination of MAVS. Thus, our findings reveal novel insights into the role of TRIM22 in RIG-I-mediated innate immunity, enhancing type I IFN production by facilitating MAVS polyubiquitination. Moreover, we observed that TRIM22 expression is critical for type I IFN responses triggered by poly(I:C), 5′-pppRNA, or IAV, which stimulate the RIG-I-mediated pathway. TRIM22 expression was significantly induced following infection with IAV or VSV, suggesting that TRIM22 forms a positive feedback loop with type I IFN, where increased IFN production enhances the expression of downstream effectors. Our results, alongside previous findings (45–47), reveal that the TRIM protein family utilizes distinct mechanisms to target different components of the RIG-I signaling pathway. This strategy could enhance the host defense system and counteract viral replication and virulence.

Ubiquitin chain types covalently attached to target proteins have emerged as key regulators of protein function. K48-linked polyubiquitination signals proteasomal degradation, while K63-linked polyubiquitination represents a non-proteolytic modification crucial for the assembly and function of protein signaling complexes (48–50). Similar to most TRIM proteins, TRIM22 contains an N-terminal RING domain and functions as an E3 ubiquitin ligase. Recent studies have shown that TRIM22 plays a key role in antiviral immunity. It promotes the degradation of viral proteins by binding to them and mediating their ubiquitination, thereby inhibiting viral replication and spread. For instance, TRIM22 has been found to inhibit influenza virus infection by ubiquitinating and degrading the viral nucleoprotein (51). Additionally, TRIM22 can limit the replication of SARS-CoV-2 by facilitating the ubiquitination and degradation of its non-structural protein NSP8 (52). Studies have also indicated that TRIM22 can suppress the replication of porcine reproductive and respiratory syndrome virus by ubiquitinating and degrading its nucleocapsid protein (53). Furthermore, TRIM22 has been shown to exert antiviral effects by ubiquitinating and degrading the 3C protease of encephalomyocarditis virus (54). These findings highlight the important role of TRIM22 in restricting various viral infections. In this study, we focus on the significant role of TRIM22 in the innate immune pathway, further supporting the hypothesis that TRIM22 may possess broad-spectrum antiviral activity. Intriguingly, TRIM22 utilizes ubiquitination in the antiviral immune response to RNA virus infection: K63-linked polyubiquitination of MAVS promotes the activation of the RIG-I signaling cascade and the production of type I IFN. Further investigation into the molecular mechanisms and clinical relevance of TRIM proteins is expected to yield valuable insights for their potential therapeutic applications (43, 55, 56).

We also discovered that ELF3 activates transcription at TRIM22 promoters, thereby increasing TRIM22 expression. As a member of the ETS family, ELF3 shares fundamental functions with other members, such as DNA binding and gene regulation, but differs in tissue specificity, cancer association, and the regulation of inflammation and immunity (12, 57). Post-RNA virus infection, we observed increased nuclear translocation of ELF3, which enhances TRIM22 expression.

NLRX1, a modulator of PAMP receptors, is generally considered a negative regulator of the innate immune response to viral infections (25, 58–62). However, some studies present alternative perspectives, suggesting a more complex role for NLRX1 in immune regulation (63–65). Studies have shown that NLRX1 exerts opposing effects on the RNA virus-induced activation of two members of the IRF family. NLRX1 is essential for the maximal induction of IRF1 expression, yet it inhibits IRF3 activation (66). As pivotal regulators of cytokine transcription, IRF family members are activated by various stimuli across different cell types (67). This complexity in IRF activation allows for a diverse array of responses, crucial for effective host defense (68–72). The contradictory regulatory roles of NLRX1 on IRF3 and IRF1 responses may explain the ongoing debate surrounding this protein. Interestingly, our findings indicate that TRIM22 can attenuate the inhibitory effects of NLRX1 on innate immunity, providing an explanation for the conflicting roles of NLRX1 within the IRF family, a discovery that holds significant implications. NLRX1 localizes to the mitochondrial outer membrane and interacts with MAVS, disrupting virus-induced IFN-β signaling (13, 73). MAVS, as a mitochondrial membrane protein, serves as a critical adaptor in the IFN-β signaling pathway, linking viral RNA recognition to downstream signal activation such as IRF3 in antiviral responses (19, 74). We find that TRIM22 promotes the dissociation of MAVS from NLRX1, alleviating the inhibitory effect of NLRX1 on MAVS.

**Fig 7:**
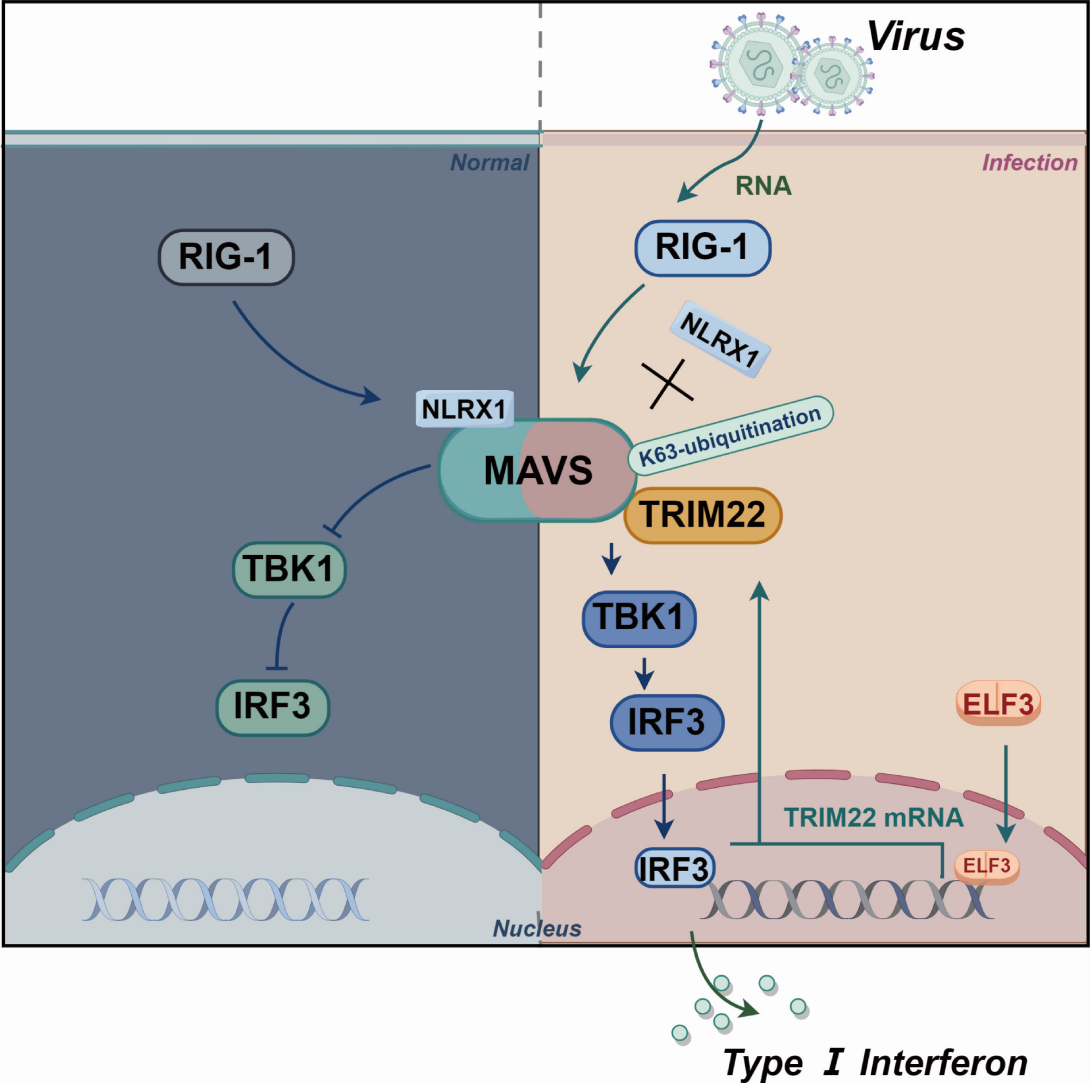
Schematic representation of the role of TRIM22 in antiviral immunity. ELF3, TRIM22, and MAVS form a feedback loop in which ELF3 translocates to the nucleus following RNA virus infection, leading to the transcriptional regulation of TRIM22 expression. Subsequently, TRIM22 catalyzes K63-linked polyubiquitination of MAVS, activating the RIG-I antiviral signaling pathway and promoting the dissociation of MAVS from NLRX1.

In conclusion, we demonstrate that ELF3, TRIM22, and MAVS form a regulatory loop (Fig. 7). In this loop, ELF3 rapidly translocates to the nucleus to activate TRIM22 gene expression after viral infection, while TRIM22 activates IFN-β antiviral signaling through K63-linked polyubiquitination of MAVS. While our study provides valuable insights into the role of TRIM22 in regulating MAVS-mediated antiviral immunity, it would be meaningful to extend the analysis to additional RNA virus strains and even DNA viruses to further explore the generalizability of our findings. The absence of TRIM22 in mice poses a barrier to further understanding the TRIM22-MAVS interaction, and the use of humanized mice may help overcome this limitation. Leveraging this natural defense mechanism may provide more effective strategies for controlling virus infections.

## MATERIALS AND METHODS

### Antibodies and reagents

5′-pppRNA and poly(I:C) were purchased from InvivoGen and were used at final concentrations of 0.5 µg/mL and 10 µg/mL, respectively. The IFN-β ELISA kit and the Dual-Luciferase Reporter Assay System were purchased from Sigma Aldrich. Anti-TRIM22 (NBP1-81795) was obtained from Novus. Anti-MAVS (24930), anti-TBK1 (3504), anti-p-TBK1 (5483), anti-IRF3 (4302), anti-p-IRF3 (4947), anti-K63-linkage specific polyubiquitin (5621), anti-K48-linkage specific polyubiquitin (8081), mouse anti-rabbit IgG (light-chain specific) (93702), and goat anti-mouse IgG (Light-Chain Specific) (91196), anti-NLRX1 (13829) were sourced from Cell Signaling Technology. Anti-Flag (20543-1-AP), anti-His (66005-1-Ig), anti-PCNA (10205-2-AP), and anti-GAPDH (60004-1-Ig) were from Proteintech. Mouse anti-NP antibody was purchased from Millipore. Mouse IgG (A7028) was from Beyotime. Alexa Fluor 633 goat anti-mouse IgG (H + L) (red, A21050) and Alexa Fluor 488 donkey anti-rabbit IgG (H + L) (green, A21206) from Life Technologies were used for fluorescence assay. Cells and viruses HEK293T, A549, BEAS-2B, and MDCK cells were cultured in DMEM (Life Technologies) containing 10% fetal bovine serum (Sigma-Aldrich). IAV (H1N1, H3N2), VSV, and VSV-GFP virus were stored in our laboratory.

### Plasmids and transfections

TRIM22 plasmids were purchased from GenePharma, amplified by standard PCR, and cloned into pcDNA3.1 with a flag tag at the N-terminus. All plasmid constructs were confirmed by DNA sequencing. SiRNA sequences for the transient silencing of human ELF3 (siRNA#1, sense: GCUGCAACCUGUGAGAUUATT, antisense: UAAUCUCACAGGUUGCAGCTT; siRNA#2, sense: CCUCUGCAAUUGUGCCCUUTT, antisense: AAGGGCACAAUUGCAGAGGTT) were obtained from GenePharma. For transient transfection of plasmids or siRNA duplexes into A549 and HEK293T cells, Lipofectamine 3000 reagent (Invitrogen) was used according to the manufacturer’s instructions. HEK293T cells were transfected with TRIM22-shRNA or overexpression plasmids along with psPAX2 and pMD.2G plasmids (Addgene) using the Hieff Trans Liposomal Transfection Reagent (YEASEN) to generate lentiviruses. Lentiviruses were collected after 48 h and used to infect target cells. Infected cells were selected with puromycin (Solarbio) 48 h post-infection to establish stably transfected cell lines.

### Plasmid sequences and lentiviral vectors

Lentiviral vectors were generated using the pLKO.1-PuroR plasmid for constructing knockdown plasmids of TRIM22. The shRNA sequences targeting TRIM22 (shRNA#1 sequence: CCGGTATTGGTGTTCAAGACTATATCTCGAGATATAGTCTTGAACACCAATATTTTTG; shRNA#2 sequence: GCATAAACGAGGTGGTCAA) were integrated into the lentivirus backbone plasmid. The negative control (NC) shRNA was purchased from Tsingke Biotech. The pLV3-CMV-PuroR vector was used to construct an overexpression plasmid for the full-length or mutated forms of MAVS (NM_020746), TRIM22 (NM_006074), and an empty vector plasmid.

### Luciferase reporter assay

The IFN-β promoter luciferase reporter plasmid was stored in the laboratory. The TRIM22 promoter sequence was cloned into a firefly luciferase reporter pGL3 vector, with the co-transfected pRL-TK Renilla luciferase plasmid serving as an internal control. Luciferase activity was measured using the Dual-Luciferase Reporter Assay System (Promega) and quantified with an Infinite M200 Pro microplate reader (Tecan). The relative luciferase activity was normalized by calculating the ratio of firefly luciferase to Renilla luciferase.

### Ubiquitination assay

For analysis of the MAVS ubiquitination in HEK293T cells, HEK293T cells were transfected with plasmids expressing His–MAVS, HA–ubiquitin (WT), HA–ubiquitin (K48O), HA–ubiquitin (K63O), and Flag–TRIM22 (WT) or its mutants. Whole-cell extracts were then immunoprecipitated with specific antibodies and analyzed by immunoblotting with anti-ubiquitin, anti-ubiquitin (K48), or anti-ubiquitin (K63).

### Immunofluorescence assay

Cells were fixed with 4% (wt/vol) paraformaldehyde in phosphate-buffered saline (PBS) for 20 min. The permeabilized cells were blocked with 5% bovine serum albumin in PBS for 1 h, followed by staining with the indicated primary antibodies and incubation with secondary antibodies conjugated to Alexa Fluor 488 or Alexa Fluor 633. Nuclei were counterstained with DAPI (Sigma-Aldrich).

### Enzyme-linked immunosorbent assay

The concentration of IFN-β in culture supernatants was measured using ELISA Kits (R&D Systems).

### Western blot and co-immunoprecipitation

Total proteins were lysed, separated by SDS-PAGE, and transferred onto polyvinylidene fluoride (PVDF) membranes (Millipore). Membranes were blocked with 5% skim milk and incubated with primary and secondary antibodies. Protein bands were detected using the ECL kit (P10300, NCM Biotech). For Co-IP, lysates were incubated with specific antibodies overnight, followed by incubation with Protein A/G agarose beads (HY-K0202, MCE). The beads were washed, and eluates were separated by SDS-PAGE. The “IP” sample refers to the protein samples obtained after washing and eluting the immunoprecipitates, whereas the “Input” sample refers to the cell lysate fraction prior to enrichment with agarose beads. GAPDH was used as an internal control.

### RNA quantification

Total RNA was extracted using TRIzol reagent (Invitrogen) and was used to synthesize first-strand cDNA with a cDNA synthesis kit (Takara). Specific primers used for real-time PCR are shown in Table 1. Real-time PCR was performed using the SYBR Green PCR Master Mix (Takara) with a LightCycler 480 II system (Roche). Relative RNA quantities were determined using the 2^−ΔΔCt^ method and were normalized to calculate relative expression changes.

**TABLE 1:** Primer sequences used for real-time qPCR analysis

Gene	Species	Sequence (forward)	Sequence (reverse)
TRIM22	Human	CTGTCCTGTGTGTCAGACCAG	TGTGGGCTCATCTTGACCTCT
TRIM15	Human	AGGAGCACGGCGAGAAGAT	GATCCCGGTAGGGCTGAATG
TRIM25	Human	AGGGATGAGTTCGAGTTTCTGG	GTTTTTGAGGTCTATGGTGCTCT
TRIM31	Human	AACCTGTCACCATCGACTGTG	TGATTGCGTTCTTCCTTACGG
TRIM69	Human	CTTGCCATCCAACAGGGTCAA	TTCCTTGTGAGCAGCAATAGC
IAV-NP	Human	AACTTGAACAGCACATTCAGAC	TTTTTCCCCTCGTTTGCTTTAG
ELF3	Human	GCCATTGACTTCTCACGATGT	GAGGCCCAAAGACCAGACG
IFN-β	Human	CGAGACACATCCAATGACCCTGAAC	GGCGGACAGCATAGGCAAGAAG
MAVS	Human	TTCTAATGCGCTCACCAATCC	CCATGCTAGTAGGCACTTTGGA
NLRX1	Human	CAGCGACCAGATGATCGTATC	TGGTGGCGTATAAAGGCCCTA
GAPDH	Human	GGAGCGAGATCCCTCCAAAAT	GGCTGTTGTCATACTTCTCATGG

### Viral infection *in vitro*

For experiments involving virus infection, A549 or HEK293T cells were infected with SeV at an MOI of 0.1 for the indicated periods of time. The cell supernatants were collected and inactivated with ultraviolet light. VSV-GFP replication was analyzed by fluorescence microscopy. A549 and HEK293T cells were also infected with the IAV (H1N1 or H3N2) virus at an MOI of 0.1.

### Virus copy number assessment

To quantify viral copies in cells, 10-fold serial dilutions of the pcDNA3.1-NP plasmid containing the viral protein (ranging from 1 × 10^1^ to 1 × 10^8^ template copies per reaction) were analyzed by qPCR to generate a standard curve based on cycle threshold (CT) values for each dilution. Total viral copies were then calculated from the CT values using the resulting standard curve. Additionally, the viral load of each sample was estimated and expressed as log10 viral copies per microliter.

### Chromatin immunoprecipitation

The ChIP assay was performed using the SimpleChIP Plus Enzymatic Chromatin IP Kit (9005, CST) following the manufacturer’s protocol. Fragmented DNA was incubated overnight at 4°C with antibodies against ELF3 (MA5-35683, Invitrogen) or normal rabbit IgG (2729, CST) as a control. The antibody-protein-DNA complexes were then captured with ChIP-grade Protein G magnetic beads and incubated overnight at 4°C with rotation. The DNA was subsequently eluted, de-crosslinked, purified, and quantified by real-time quantitative reverse transcription-PCR (RT-qPCR). ChIP primers for the TRIM22 promoter were designed as follows: forward 5′-AGCTTATTATTCTGTGAGCAACAAA-3′; reverse 5′-ACATGGATCCCTGACTGACAC-3′.

### Statistical analysis

Data were statistically analyzed using a two-tailed unpaired Student’s *t*-test with GraphPad Prism 9 software. A *P*-value of <0.05 was considered statistically significant.

## Data Availability

All data are included in the paper or are available from the authors upon reasonable request. Correspondence and material requests should be addressed to Xiaoshen Zhang.
